# Multiple Functions of the *Dmrt* Genes in the Development of the Central Nervous System

**DOI:** 10.3389/fnins.2021.789583

**Published:** 2021-12-09

**Authors:** Takako Kikkawa, Noriko Osumi

**Affiliations:** Department of Developmental Neuroscience, United Centers for Advanced Research and Translational Medicine (ART), Tohoku University Graduate School of Medicine, Sendai, Japan

**Keywords:** *DmrtA* subfamily, patterning, neurogenesis, neuronal specification, corticogenesis

## Abstract

The *Dmrt* genes encode the transcription factor containing the DM (doublesex and mab-3) domain, an intertwined zinc finger-like DNA binding module. While *Dmrt* genes are mainly involved in the sexual development of various species, recent studies have revealed that *Dmrt* genes, which belong to *the DmrtA* subfamily, are differentially expressed in the embryonic brain and spinal cord and are essential for the development of the central nervous system. Herein, we summarize recent studies that reveal the multiple functions of the *Dmrt* genes in various aspects of vertebrate neural development, including brain patterning, neurogenesis, and the specification of neurons.

## Introduction

The *Dmrt* (*double-sex* and *mab-3* related transcription factor) genes encode a large family of transcription factors involved in sexual development (Zarkower, 2001; Kopp, 2012). First identified as Doublesex in *Drosophila* and MAB-3 in *Caenorhabditis elegans* (*C. elegans*), the Dmrt family proteins share a DM (doublesex and mab-3) domain that consists of a highly intertwined zinc finger DNA-binding motif (Erdman and Burtis, 1993; Raymond et al., 1998). Several *Dmrt* genes have been identified in vertebrates, including Dmrt1 to Dmrt8 in mice and humans (Bellefroid et al., 2013). Among these, the *DmrtA* subfamily members (DmrtAs, i.e., Dmrt3, Dmrta1, and Dmrta2) have a conserved DMA domain near the C-terminus in addition to the DM domain (Ottolenghi et al., 2002). It has been reported that the DMA domain is required for the activity of the Dmrt protein (Parlier et al., 2013). Moreover, the DMA domain in DMD-4 in *C. elegans* can bind to ubiquitin and stabilize the protein, thus playing a sex-specific role in synaptic connectivity (Bayer et al., 2020). Although each Dmrt acts as a transcriptional regulator, the DM domain proteins can form heterodimers on DNA, raising the possibility of combinatorial gene regulation by these proteins (Murphy et al., 2007).

In vertebrates, Dmrt-family genes are primarily involved in the development of sexual organs. As a representative function, Dmrt1 controls many aspects of testicular development, including the postnatal differentiation of germ cells and Sertoli cells (Raymond et al., 2000; Kim et al., 2007a). Dmrt7 localizes to spermatocytes, specifically the XY body, a domain where X and Y chromosomes are silenced and compartmentalized, which leads to the proper meiotic progression in the mouse testis (Kawamata and Nishimori, 2006; Kim et al., 2007b). In addition to the development of sexual organs, the Dmrt factors are involved in various events during embryonic development (Hong et al., 2007; Bellefroid et al., 2013). For example, Dmrt2 is necessary for the establishment of left-right asymmetry during somitogenesis (Saude et al., 2005; Liu et al., 2009; Lourenco et al., 2010). Based on recent findings, including ours, this review focuses on various functions of *Dmrt* genes, especially *DmrtA* subfamily members, in central nervous system (CNS) development.

## Expression Patterns of *Dmrt* Genes in the Developing Central Nervous System

**FIGURE 1:**
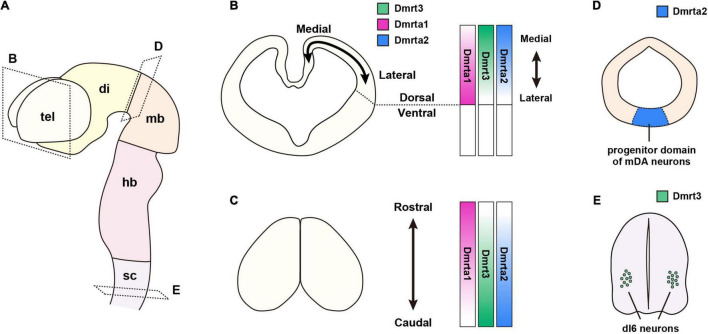
Expression patterns of DmrtA subfamily members in the embryonic mouse CNS. **(A)** Schematic representation of the mouse embryonic CNS at E11. **(B–E)** Expression pattern of DmrtAs. **(B)** Cross-sections of the telencephalon. Magenta indicates the expression of Dmrta1; green, Dmrt3; and blue, Dmrta2. DmrtAs are expressed in NSPCs in the dorsal telencephalon. Dmrta1 expression has a medial-low/lateral-high gradient, whereas Dmrt3 and Dmrta2 show a medial-high/lateral-low gradient. **(C)** Dorsal view of the telencephalon. Dmrta1 is expressed in a rostral-high/caudal-low gradient, whereas Dmrt3 and Dmrta2 show a rostral-low/caudal-high gradient. **(D)** Cross-sections of the midbrain. Dmrta2 is expressed in the progenitor domain of mDA neurons in the ventral midbrain. **(E)** Cross-sections of the spinal cord. Dmrt3 is specifically expressed in dI6 neurons in the spinal cord. CNS, central nervous system; di, diencephalon; dI, dorsal interneuron; DmrtAs, DmrtA subfamily members; hb, hindbrain; mb, midbrain; NSPCs, neural stem progenitor cells; sc, spinal cord; tel, telencephalon.

In various regions of the developing brain, DmrtAs are mainly expressed in neural stem/progenitor cells (NSPCs) in the ventricular zone (VZ), which lines the ventricular wall. In the spinal cord, the expression of DmrtAs are detected in specific neurons. We summarize the expression patterns of DmrtAs in each region of the CNS during development (Figure 1).

In the mouse telencephalon, mRNAs of *Dmrt3*, *Dmrta1*, and *Dmrta2* are detected as early as embryonic day (E) 9.5, and their levels peak around E10.5–E12.5 (Konno et al., 2012). The expression of *Dmrta1* in rats begins on E10.5 (corresponding to E8.5), an early stage of CNS development (Kikkawa et al., 2013). In non-rodent species, chick embryos begin to show a strong expression of *Dmrt3* in the developing telencephalon by E2.5 (Smith et al., 2002). *Xenopus Dmrta1 (XDmrt4)* is initially expressed in the anterior neural ridge and is restricted to a part of the telencephalon by stage 35 (Huang et al., 2005). Medaka fish (*Oryzias latipes*) also show restricted expression in the dorsal telencephalon at stage 26, and the mRNA level gradually decreases thereafter (Winkler et al., 2004). *Ciona*, a member of the vertebrate sister group Urochordata, expresses *Dmrt1* (related to Dmrta1 and Dmrta2 and with a DMA domain) in the anterior neural plate and is later restricted it to the anterior brain at the tailbud stage (Bellefroid et al., 2013). Thus, the expression of DmrtAs in the telencephalon appears to be conserved across species.

Detailed and differential expression patterns of DmrtAs in the telencephalon have been studied in mice. Focusing on the dorsoventral and mediolateral axes of the telencephalon, *Dmrt3* and *Dmrta2* are expressed with a medial-high/lateral-low gradient (Konno et al., 2012, 2019; Kikkawa et al., 2013), whereas Dmrta1 has a medial-low/lateral-high gradient in the dorsal telencephalon (Kikkawa et al., 2020 and our unpublished data; Figure 1B). In the rostral-caudal axis, *Dmrt3* and *Dmrta2* show a rostral-low/caudal-high gradient (Figure 1C). Conversely, *Dmrta1* has a relatively rostral-high/caudal-low expression level (Kikkawa et al., 2020). Their functional differences will be discussed later in the sections on “brain patterning” and “corticogenesis.”

In the diencephalon, another structure subdivided from the forebrain, the expression pattern of the *Dmrt* gene is different from that in the neighboring telencephalon. In zebrafish, *Dmrta2* is restricted to the ventral region of the diencephalon and hypothalamus (a part of the diencephalon) at the 6-somite stage (Guo et al., 2004; Yoshizawa et al., 2011). *Xenopus* also shows *Dmrta2* expression in the ventral diencephalon as does the zebrafish (Parlier et al., 2013).

*Dmrta2* expression in the mesencephalon (midbrain) is restricted ventrally, as in the diencephalon (Figure 1D). This expression pattern is found in platyfish, chicks, and mouse embryos (Veith et al., 2006; Gennet et al., 2011; Saulnier et al., 2013); however, it appears not to be the case in *Xenopus* (Parlier et al., 2013). In *Dmrta1*, our *lacZ* knock-in mice to the locus of *Dmrta1* show β-galactosidase (β-gal) expression in the ventral midbrain (Kikkawa et al., 2020). The ventral midbrain is the primary origin of midbrain dopaminergic (mDA) neurons (Gale and Li, 2008). Interestingly, Dmrta2 is a regulator of the mouse ventral mesencephalic neural fate specification (Gennet et al., 2011) (see below).

Only Dmrt3 is expressed in the spinal cord. Unlike in brain regions, it is expressed in differentiated neurons but not in NSPCs. *Dmrt3* expression in the embryonic spinal cord appears to be conserved among mice, chicks, and fish (Smith et al., 2002; Kim et al., 2003; Winkler et al., 2004; Li et al., 2008). Mouse embryonic data show that Dmrt3 is specifically expressed in the dorsal interneuron (dI) subtype, dI6 neurons, in the spinal cord and regulates their neuronal specification of the interneuron subpopulation (Andersson et al., 2012; Figure 1E) (see below).

As described above, DmrtAs are expressed during the early embryonic development of the CNS in various species. We discuss the function of DmrtAs in brain development in the following sections.

## Functions of *Dmrt* Genes in Brain Patterning

The telencephalon is patterned by the combined action of different signaling centers, such as the rostral signaling center in the rostromedial forebrain secreting Fgfs, the dorsal cortical hem (CH) in the caudomedial telencephalon secreting Wnts and BMPs, and in the floor plate at the ventral midline of the embryonic forebrain secreting Sonic hedgehog (Shh) (Hebert and Fishell, 2008). These factors specifically regulate the transcription of target genes, thus defining the specific fate of cells and conferring positional information along the axis (Wilson and Houart, 2004; Rhinn et al., 2006; Monuki, 2007).

**FIGURE 2:**
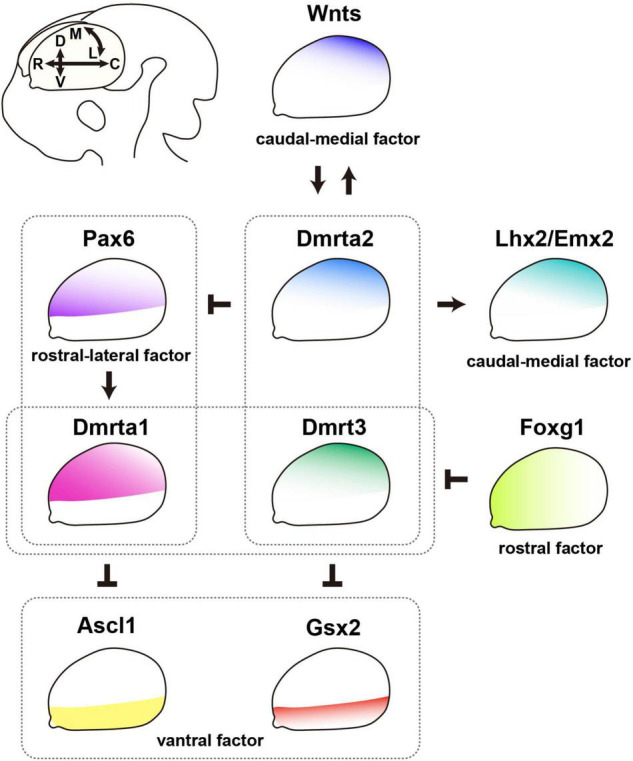
DmrtA subfamily members contribute to the patterning of the telencephalon. Regulatory networks of telencephalic patterning molecules related to the contribution of DmrtA subfamily members (DmrtAs). DmrtAs are involved in subdivision along the dorsoventral axis of the telencephalon. Dmrta1 is a direct target of the transcription factor Pax6, which exhibits a rostral-high/lateral-high gradient expression. Dmrta1 represses Ascl1 expression in the ventral telencephalon, while Dmrt3 and Dmrta2 directly repress the expression of *Gsx2*. Therefore, DmrtAs are essential for determining the dorsoventral identity of progenitor cells by repressing ventralization. DmrtAs play roles in mediolateral patterning of the dorsal telencephalon. The caudal-medial signaling center secretes Wnts that are required for the formation of the medial telencephalic structure. Wnts and DmrtAs mutually regulate each other. Dmrt3 and Dmrta2 mainly determine the proper patterning of the caudal-medial telencephalon. Dmrta1 supports medial telencephalic development. The rostral factor Foxg1 represses *Dmrt3* and *Dmrta1* expression, and this molecular pathway may be needed to acquire the caudal-medial fate specification. Furthermore, Dmrta2 modulates the expression of transcription factors such as *Pax6*, *Emx2*, and *Lhx2*, leading to the proper patterning of the neocortex. C, caudal region; D, dorsal region; L, lateral region; M, medial region; R, rostral region; V, ventral region.

Since DmrtAs are strongly expressed in the telencephalon, many recent studies have reported their involvement in regionalization (Figure 2). Therefore, we would like to mention the roles of DmrtAs in telencephalic patterning, including the upstream and downstream molecular networks of DmrtAs.

### Subdivisions Along the Dorsoventral Axis of the Telencephalon

The telencephalon is patterned into two major subdivisions, the dorsal and ventral regions, called the pallium and subpallium, respectively (Puelles et al., 2000). NSPCs in the pallium produce excitatory neurons, whereas those in the subpallium produce interneurons (Anderson et al., 1997; Gorski et al., 2002). For proper patterning along the dorsoventral (DV) axis, it is essential that several transcription factors work in concert. For example, the loss of Pax6, a transcription factor expressed in the dorsal region, shows severe ventralization with a dorsal expansion of the expression of Gsx2, Ascl1, and Dlx1, which are involved in the production of interneurons from the ventral region; thus, Pax6 is crucial for patterning the dorsal and ventral telencephalon (Stoykova et al., 2000; Toresson et al., 2000). In particular, the positioning of the DV boundary region is defined by Pax6-Gsx2 mutual antagonism (Corbin et al., 2000; Toresson et al., 2000; Yun et al., 2001).

DmrtAs are also crucial for the determination of the DV axis. Recent *Dmrt3* and *Dmrta2* knock-out (KO) studies have clarified how they specify DV regional identity in progenitors as follows. *Dmrt3;Dmrta2* double-KO (dKO) mice showed the most severe phenotype of DV regionalization of the telencephalon than *Dmrta2* single-KO (sKO) mice, and *Dmrt3* sKO mice showed the mildest phenotype (Desmaris et al., 2018; Konno et al., 2019). It is noteworthy that the expression of subpallium-specific markers (e.g., *Gsx2*, *Dlx2*, *Ascl1*) is shifted dorsally to the lateral part of the dorsal telencephalon in *Dmrt3;Dmrta2* dKO mice (Desmaris et al., 2018). Furthermore, although *Dmrta1* sKO mice did not have a phenotype of ventralization, the overexpression of *Dmrta1* in the ventral telencephalon in rats induced the expression of the dorsal marker Neurog2 and repressed that of the ventral marker Ascl1 (Kikkawa et al., 2013). These results indicate that DmrtAs function as an essential determinant of progenitor cell DV identity by repressing ventralization.

Therefore, a question arises: what are the main target genes of DmrtA transcription factors that determine telencephalic identity? The enhancer activity of the *Gsx2* locus, to which Dmrta2 binds, is found in the ventral telencephalon (Desmaris et al., 2018). A more comprehensive method, whole-genome chromatin immunoprecipitation-sequencing (ChIP-seq), revealed that Dmrt3 and Dmrta2 bind to the *Gsx2* and *Pax6* loci within ±100 kb from the transcription start site (Konno et al., 2019). The authors also generated transgenic mice to visualize the enhancer activity of Dmrt3/Dmrta2-binding sites at *Gsx2* and *Pax6*. They found that the enhancer activities of the loci at *Gsx2* and *Pax6* bound by Dmrts are detected in the dorsal lateral ganglionic eminence (dLGE) and the dorsal telencephalon, where Gsx2 and Pax6 are expressed, respectively. Interestingly, a suppressive histone moiety, H3K27me3, is found in the Gsx2 enhancer bound by Dmrt3/Dmrta2 in their study, suggesting that DmrtAs may suppress the expression of Gsx2. Since the dLGE neighboring the DV boundary expresses a high level of Gsx2, its direct regulation may modulate the fate specification of the progenitors in the dorsal and ventral telencephalon.

Pax6 regulates the expression of various genes in the embryonic telencephalon (Sansom et al., 2009; Kikkawa et al., 2013, 2019; Walcher et al., 2013; Xie et al., 2013; Sun et al., 2015). We initially searched for downstream genes of Pax6 by comparing the transcriptomic profiles of telencephalic samples from wild-type and *Pax6* null mutant rat embryos and found *Dmrta1* as a novel target (Kikkawa et al., 2013) in addition to the various downstream molecules in the embryonic CNS (Shimoda et al., 2002; Arai et al., 2005; Numayama-Tsuruta et al., 2010; Shinohara et al., 2013; Inada et al., 2018). Pax6 ChIP-seq has later proved the direct regulation of the *Dmrta1* gene by the Pax6 transcription factor in the E12.5 mouse forebrain (Sun et al., 2015). However, this Pax6-Dmrta1 pathway seems to work only in specific dorsal-lateral telencephalic regions. Dmrta1 expression was markedly decreased in the neocortex of *Pax6* homozygous mutant rat embryos, although it remained in the CH (Kikkawa et al., 2013). Focusing on the functions in the formation of the CH, Dmrta1 supports the generation of the CH (Kikkawa et al., 2020), whereas Pax6 suppresses its fate specification of the CH (Godbole et al., 2017). Therefore, the Pax6-Dmrta1 pathway may work differently in the neocortex and extra-neocortical areas, such as the CH.

### Patterning of the Dorsal Telencephalon Along the Mediolateral Axis

The dorsal telencephalon is divided into the neocortex, hippocampus, and dorsal midline, giving rise to the CH and choroid plexus. The dorsal midline of the telencephalon is known to express BMPs and Wnts (Furuta et al., 1997; Grove et al., 1998). BMP and Wnt signaling are required for the formation of the medial telencephalic structure that develops into the hippocampus and the dorsal midline region (Lee et al., 2000; Hebert et al., 2002), and both signaling pathways also regulate the expression of transcription factors such as Emx2 and Lhx2, which specify and expand the medial and dorsal parts of the telencephalon (Monuki et al., 2001; Theil et al., 2002). These molecular pathways are essential for patterning the dorsal telencephalon along the mediolateral axis.

In addition to these critical molecules that regulate the development of the medial telencephalon, it has become clear that DmrtAs contribute to the following events. Previous studies have demonstrated malformation of caudomedial telencephalic structures, that is, the CH that expresses *Wnt3a* and *Bmp4*, and the hippocampus, in *Dmrta2* sKO mice (Konno et al., 2012; Saulnier et al., 2013; De Clercq et al., 2018). *Dmrt3* sKO embryos also have shown defects in the CH formation (De Clercq et al., 2018; Kikkawa et al., 2020). These phenotypes are reasonable because Dmrt3 and Dmrta2 are expressed with medial-high/lateral-low and rostral-low/caudal-high gradients. Although the expression level of Dmrta1 was weak in the medial telencephalon, *Dmrt3;Dmrta1* dKO mice exhibited more severe defects in medial structures compared to *Dmrt3* sKO mice (Kikkawa et al., 2020). Therefore, Dmrta1 may have an additional support function for patterning medial telencephalic structures in cooperation with Dmrt3.

When do the defects in the medial telencephalon of *Dmrt* mutants begin? It has been reported that DmrtAs are expressed in the forebrain at early developmental stages, that is, E10.5–E12.5 (Konno et al., 2012; Kikkawa et al., 2013). Because *Wnt3a^Cre^*-driven *Dmrta2* conditional KO (cKO) mice showed normal morphology of the medial telencephalon (De Clercq et al., 2018), the apparent defects in the medial telencephalon by the ablation of *DmrtAs* may be due to the loss of their expression during earlier developmental stages before the initiation of recombination by *Wnt3a-Cre* at E10 in mice (Yoshida et al., 2006). These findings suggest that *DmrtAs* establish the dorsal midline structure, a signaling center that expresses Wnt and BMP in patterning the cerebral cortex.

Then, what molecules function upstream of DmrtAs to determine the dorsomedial patterning of the telencephalon? *Dmrt* gene expression was downregulated in *Gli* mutant mice, in which *Wnt* expression in the forebrain was severely affected (Hasenpusch-Theil et al., 2012). This study also clarifies that *Dmrt3* is a direct Wnt target gene in the dorsomedial telencephalon by DNA binding and reporter gene assays. Furthermore, the expression of Dmrt3 and Dmrta2 were decreased by the overexpression of a dominant-negative form of Tcf3 and increased by the overexpression of a constitutively active form of β-catenin (Konno et al., 2012). Moreover, *Dmrta2* expression was induced in organotypic slice cultures of mouse embryonic telencephalons treated with Chir that selectively inhibits GSK3β and activates Wnt signaling (Saulnier et al., 2013). This means that the secretory factor Wnts can directly regulate *Dmrt* expression and could contribute to adjusting the amount of *Dmrts*.

### Arealization of the Neocortex

The neocortex has “primary” areas: the primary motor (M1, controls the voluntary movement of body parts), somatosensory (S1, processes the information received from the body), and visual (V1, processes the information received from eyes) areas. One of the hypotheses to determine the cortical arealization is the “protomap model.” The feature of progenitors in early development is predetermined by the combination of molecules, and then the progenitors differentiate into specific neurons, leading to the formation of different cortical regions (Rakic, 1988). Some transcription factors that contribute to the formation of the “protomap.” Rostral*^high^*-Pax6 and caudal*^high^*-Emx2 in progenitors preferentially impart the identities of the rostrolateral and caudomedial areas, respectively (Bishop et al., 2002). A study on the loss of *Pax6*/*Emx2* indicates that they suppress each other’s expression (Muzio et al., 2002). Lhx2 is expressed in a graded manner (caudal*^high^*/medial*^high^*) in the telencephalon (Nakagawa et al., 1999; Monuki et al., 2001), acts as an essential determinant of cortical identity (Mangale et al., 2008), and is further required for the neocortical-paleocortical subdivision (Chou et al., 2009). *Pax6* expression is decreased in the *Lhx2* mutant in the dorsal telencephalon due to direct regulation by Lhx2 (Hou et al., 2013; Shetty et al., 2013). The combinatorial expression patterns of these transcription factors regulate the regionalization of the cerebral cortex.

Since the loss of CH affects cortical size and patterning (Caronia-Brown et al., 2014), the reduction in cortical size in *Dmrt* mutants may be due to the developmental impairment of CH formation. However, deleting Dmrta2 after CH formation still decreases the cortical size and changes the area map; the V1 area, which is the caudomedial neocortical region, was reduced in *Emx1*^Cre^*-* and *Nestin*^Cre^*-*driven *Dmrta2* cKO mice on postnatal day 7 (De Clercq et al., 2018). Conversely, *Dmrta2*^Tg/+^*; Emx1*^Cre^** mice with excess Dmrta2 in the cortical primordium showed enlarged V1 and reduced size of the S1 and M1 areas. They also revealed a decrease in *Lhx2* and *Emx2* and the expansion of *Pax6* to the caudal cortex in *Dmrta2* cKO embryos. From these results, the level of Dmrta2 seems to regulate the expression of these transcription factors that determine the rostral-caudal patterning during cortical development and construct the proper neocortical area map.

## Functions of *Dmrt* Genes in Corticogenesis

### Maintenance of NSPCs and Their Differentiation Into Neurons

NSPCs maintain their population by self-renewal and produce neurons by differentiation during embryogenesis. At an early stage of development, that is, E9–E11 in the mouse telencephalon, NSPCs undergo symmetric division in the VZ, producing daughter cells with similar fates to give rise to more progenitors. As development proceeds, NSPCs start to divide asymmetrically, producing one apical progenitor (AP) positive for Pax6 with self-renewing capability and one differentiated neuron, or one intermediate progenitor (IP) positive for Tbr2, which divides symmetrically in the subventricular zone (SVZ) and generates a pair of IPs or neurons (Gotz and Huttner, 2005; Huttner and Kosodo, 2005). Subsequently, newly born neurons migrate radially from the VZ/SVZ to the upper area of the telencephalon. In this section, we introduce the multiple roles of DmrtAs expressed in NSPCs in the telencephalon in cell proliferation and differentiation regulation.

Dmrta2 is expressed in NSPCs derived from mouse embryonic stem cells (ESCs) (Young et al., 2017). It maintains their proliferation by positively regulating *Hes1* expression *via* Dmrta2 bound to the locus of *Hes1* (Young et al., 2017), a significant Notch target gene that inhibits neuronal differentiation through negative regulation of proneural genes (Kageyama et al., 2020). Consistently, the neurogenic gene *Neurog2* was upregulated in the medial region of the dorsal telencephalon of *Dmrta2* sKO mice (Saulnier et al., 2013). Furthermore, in *Dmrt3*, transgenic mice overexpressing Dmrt3 in NSPCs under the control of the *Nestin* enhancer showed a planar expansion of the ventricular surface, indicating an increased NSPC pool and decreased Tbr2-positive IP cells (Konno et al., 2019). These results suggest that Dmrt3 and Dmrta2 are involved in the maintenance of NSPCs in the dorsal telencephalon.

In contrast, DmrtAs seem to contribute to neuronal differentiation. A zebrafish mutant of the *ha2* locus, encoding *Dmrta2*, reduced the expression of *neurog1* and impaired telencephalic neurogenesis (Yoshizawa et al., 2011). In addition to the brain, *Xenopus* Dmrta1 and Dmrta2 promoted neurogenesis in the olfactory placode (Huang et al., 2005; Parlier et al., 2013). Although Dmrt3 and Dmrta2 are involved in the maintenance of NSPCs, as mentioned above, the ratio of Tbr2^+^ to the total number of progenitors was lower, while that of Pax6^+^ cells was higher, in *Dmrta2* sKO mice than in wild type mice in the lateral cortex (Ratie et al., 2020). This suggests that the APs could generate fewer IPs and/or that the timing of their differentiation could be delayed. Our data showed that the knockdown of *Dmrta1* or *Dmrt3* in the rat dorsal telencephalon reduces Neurog2, while the overexpression of *Dmrta1* in the ventral telencephalon induces ectopic Neurog2 expression in the restricted lateral cortex and near the DV boundary region (Kikkawa et al., 2013). Based on the above reports, the function of *DmrtAs* in neurogenesis is not simple, and its function may be altered in a region-dependent manner within the telencephalon.

### Fate Determination of NSPCs Into Either Neurons or Glial Cells

The sequential production of neurons and glia from NSPCs is a critical event during CNS development. In the mammalian neocortex, NSPCs at the early developmental stage generate neuronal cells, whereas those at the late stage generate mainly glial cells, including astrocytes and oligodendrocytes (Qian et al., 2000). The appropriate neurogenic-to-gliogenic switch in NSPCs is essential for the production of proper numbers of neurons and glia. Various molecular mechanisms have been clarified; for example, BMP, Fgf, and Notch signaling induce the cell fate switch from neuronal to glial cells (Miller and Gauthier, 2007). The transcription factor Lhx2 suppresses astrogliogenesis and promotes neurogenesis in the developing hippocampus, but not in the neocortex, indicating the spatial-specific regulation of NSPCs to neuron/glia fate specification (Subramanian et al., 2011).

There is an exciting finding that Dmrta2 is involved in the cell fate switch from neurons to astrocytes. The loss of *Dmrta2* increased the population of GFAP-positive cultured astrocytes taken from the embryonic hippocampus (Muralidharan et al., 2017). They also showed that the loss of *Lhx2* also induces astrogliogenesis, and the phenotype is rescued by the overexpression of *Dmrta2*. These results suggest that Dmrta2 and Lhx2 reciprocally regulate each other and that this pathway is involved in the neuron-glia cell-fate switch, emphasizing the novel role of Dmrta2 as a neurogenic factor. It is reasonable to assume that this phenotype is limited to the hippocampus, but not the cortex, because both Lhx2 and Dmrta2 are strongly expressed in the medial region and have already been reported to be involved in hippocampal development (Bulchand et al., 2001; Konno et al., 2012; Saulnier et al., 2013; De Clercq et al., 2018).

In the Lhx2-Dmrta2 pathway, ChIP-quantitative PCR using the embryonic hippocampus showed that Lhx2 binds to the *Dmrta2* locus, indicating that Lhx2 could directly regulate the expression of *Dmrta2* (Muralidharan et al., 2017). Interestingly, the locus bound by Dmrta2 is evolutionarily highly conserved among species. If this mechanism of the neuron-glia cell fate switch by the Lhx-Dmrta2 pathway could be conserved among different organisms, it may provide a deeper insight into the poorly understood neuron-glia switch in other species.

### Production of Early Born Neurons

It is unclear which neuronal subtypes are regulated by DmrtAs. The expression of *DmrtAs* is abundant in developmental stages when early born neurons called Cajal-Retzius (CR) cells are produced (Konno et al., 2012; Kikkawa et al., 2013). CR cells are among the first neurons to be generated (between E9.5 and E13.5) in mice (Hevner et al., 2003; Takiguchi-Hayashi et al., 2004). CR cells are generated from specific extra-neocortical regions, that is, (1) the pallial septum, which is located adjacent to a rostral signaling center in the rostromedial telencephalon; (2) the boundary between the pallium and subpallium (pallial-subpallial boundary; PSB); and (3) the CH in the caudomedial telencephalon, and later migrate tangentially over long distances from their original production sites, gradually covering the surface of the cortex (Meyer et al., 2002; Takiguchi-Hayashi et al., 2004; Bielle et al., 2005; Yoshida et al., 2006; Garcia-Moreno et al., 2007; Imayoshi et al., 2008; Gu et al., 2009; Tissir et al., 2009). CR cells appear to play critical roles in the radial migration of subsequently born cortical neurons and eventually in the laminar organization of the cortex (D’Arcangelo et al., 1995; Ogawa et al., 1995; Super et al., 2000).

We revealed the reduced production of CH-derived CR cells in *Dmrt3* sKO mice, especially in *Dmrta1* and *Dmrt3* dKO mice (Kikkawa et al., 2020). The reduction in CR cell production is consistent with the developmental impairment of the CH from which CR cells are produced. *Dmrta2* sKO mice also have defects in the CR cells (Saulnier et al., 2013). These results suggest that DmrtAs cooperatively maintain the appropriate number of CR cells derived from specific source regions by defining the patterning of the medial telencephalon. For CR cell production, one of the candidate molecules working upstream of *Dmrt3* and *Dmrta1* may be forkhead box G1 (Foxg1). *Foxg1* cKO mice (*Foxg1^tetOFoxg1^* line, repressing *Foxg1* transgene expression in the presence of doxycycline) showed an upregulated expression of *Dmrt3* and *Dmrta1* in the dorsal telencephalon (Kumamoto et al., 2013). ChIP-seq for Foxg1 revealed direct binding to the intronic sequences of *Dmrt3* upstream and *Dmrta1* downstream. Thus, the pathway by which Foxg1 represses *Dmrt3* and *Dmrta1* expression may critically affect telencephalic regionalization. This hypothesis is consistent with the converse phenotypes in the formation of CH in *Dmrt* mutants *versus Foxg1* KO mice, showing region expansion (Dou et al., 1999; Martynoga et al., 2005; Hanashima et al., 2007).

Another early born neurons are subplate (SP) neurons generated before the excitatory neurons in the cortex; that is, between E10.5 and E12.5, in the mouse (Price et al., 1997). Glutamatergic SP neurons are derived from the cortex and rostral medial telencephalic wall (Yoshida et al., 1997; Shinozaki et al., 2002; Garcia-Moreno et al., 2008; Pedraza et al., 2014). SP neurons contribute to the establishment of the initial neural circuits between the cortex and thalamus during neocortical development (McConnell et al., 1989; Ghosh et al., 1990). SP neurons are also critical for inducing the multipolar-to-bipolar transition, which leads to a faster migration mode (Ohtaka-Maruyama et al., 2018).

A recent study has demonstrated that the production of SP neurons is significantly decreased by the loss of *Dmrt3* and *Dmrta2* (Ratie et al., 2020). To clarify the time window of the effect of Dmrta2 in SP neuron formation, they disrupted *Dmrta2* in cortical progenitors from E10.5 in *Dmrta2^Lox/Lox^*;*Emx1^Cre^* mice and from E11.5 in *Dmrta2^Lox/Lox^*;*Nestin^Cre^* mice. The SP neurogenesis was only developmentally delayed in *Dmrta2^Lox/Lox^*;*Emx1^Cre^* mice and no phenotypic change was observed in *Dmrta2^Lox/Lox^*;*Nestin^Cre^* mice. Thus, the continuous expression of Dmrta2 from an earlier stage is likely necessary for the production and specification of SP neurons. Interestingly, the Dmrta2 protein is continuously detected in SP neurons, where Dmrta2 may regulate cortical neuron migration (Ratie et al., 2020).

## Functions of *Dmrt* Genes in the Specification of Neural Stem/Progenitor Cells and Neurons Outside the Developing Telencephalon

So far, we have focused mainly on neurogenesis in the telencephalon. As mentioned earlier, Dmrta2 is also localized to the ventral-medial mesencephalic NSPCs, giving rise to mDA neurons that secrete the neurotransmitter dopamine. mDA neurons are generated from E10.5 to E14.5, with neuronal birth peaking between E11 and E12 (Bayer et al., 1995). Many factors function during mDA neurogenesis both in the expansion of the NSPC pool and in the proper specification of mDA neuronal fate (Gale and Li, 2008). Intriguingly, Dmrta2 is strongly expressed from E10.5 to E12.5 in the progenitor domains and promoted the expression of Foxa2, Lmx1a, and Msx1, (Gennet et al., 2011) which are transcription factors required for the specification of ventral-medial cell identities. Although the loss of *Dmrta2* did not affect neurogenesis (Gennet et al., 2011), Dmrta2 repressed other genes in the ventral-lateral region, independent of Shh, which mainly contributes to the production of mDA neurons. In the telencephalon, a double knockdown of *Dmrt3* and *Dmrta2* did not affect the expression of *Gli1* and *Ptch1*, which are involved in the Shh signaling pathway (Konno et al., 2019). From these results, the fate specification of the progenitors by Dmrts in both the telencephalon and midbrain seems to be regulated independently of the Shh signaling pathway. Since the β-gal signal was detected in the ventral midbrain of *lacZ* knock-in mice to the locus of *Dmrta1* (Kikkawa et al., 2020), it would be interesting to know if there could be some functional overlap between Dmrta1 and Dmrta2.

A combinatorial expression of transcription factors specifies the dorsal spinal cord lineages where NSPCs differentiate into specific interneurons in the spinal cord. Dmrt3 is not expressed in NSPCs but in dI6 neurons, originating from dI6 progenitors at around E11.5 (Andersson et al., 2012). These dI6 neurons have two populations: Dmrt3- and Wt1-expressing interneurons. Their *Dmrt3* KO mice showed an increased number of Wt1^+^ neurons, suggesting a fate change in the Dmrt3^+^ population within a specific subset of dI6 neurons. Consistent with the phenotype of the altered subpopulation of interneurons in the spinal cord, the *Dmrt3* KO mice exhibited defects in the coordinated locomotor network controlling limb movements. A premature stop codon by *DMRT3* mutation in horses changes locomotion patterns, which is favorable for harnessing racing performances (Andersson et al., 2012; Perry et al., 2019). Zebrafish studies have also reported that Dmrt3-expressing neurons contribute to locomotor activity (Del Pozo et al., 2020; Satou et al., 2020). These results suggest a conserved function of Dmrt3 in the spinal cord of vertebrates.

## Perspectives on the Role of Dmrt in Human Pathogenesis

Although little is known about the function of DmrtAs in human diseases, several studies have recently shown that *DmrtAs* are involved in neurological diseases. One case report examined a consanguineous family with three confirmed siblings affected by a severe prenatal neurodevelopmental disorder characterized by severe progressive microcephaly (Urquhart et al., 2016). Their exome sequencing identified a homozygous single base pair deletion in *DMRTA2* that lead to a frameshift variant. This phenotype in microcephaly appears to be consistent with cortical malformations in *Dmrta2* KO mice as mentioned above. The second report identified *the DMRT3* enhancer as a candidate involved in the pathogenesis of spastic cerebral palsy (Kubota et al., 2018), a disease that affects the movement and posture and is caused by a genetic abnormality in 30% of cerebral palsy cases (Fahey et al., 2017). The deletion of the enhancer, including the retinoic acid (RA) receptor/retinoid X receptor (RAR/RXR) complex-binding motif, has been identified in patients with spastic cerebral palsy (Lerer et al., 2005; Vanzo et al., 2013; Kubota et al., 2018). High-throughput chromosome conformation capture (Hi-C) data revealed that this enhancer region contacts the *DMRT3* promoter (Kubota et al., 2018). Furthermore, RA stimulation upregulated the expression of *Dmrt3* in embryonal carcinoma cells (Chatagnon et al., 2015). These results suggest that the transcription of *Dmrt3* is decreased in the absence of the enhancer, leading to cerebral palsy. Many patients with spastic cerebral palsy find it difficult to move their arms and legs smoothly, possibly with impaired limb movement due to DMRT3 dysfunction in the interneurons of the spinal cord.

## Conclusion

It is gradually becoming clear that members of DmrtA transcription factors contribute to multiple aspects of CNS development; however, their targets are still poorly understood despite their common structure containing the DM domain, which is a zinc finger DNA-binding motif. Since Dmrts form heterodimers with each other, it is possible that the transcriptional regulation is not straightforward and may be a source of complexity. As the molecular structure and expression patterns of DmrtAs are evolutionarily conserved, further analyses of these transcription factors will lead to an understanding of the shared mechanism of CNS development among various species. Recently, the involvement of DMRT in the malformation of the human brain has been identified (Urquhart et al., 2016); further findings of the DmrtA subfamily in human pathogenesis may lead to the elucidation of new insights for the understanding of diseases caused by developmental abnormalities in the CNS.

## Author Contributions

TK drafted the manuscript and created the Figures. TK and NO discussed and revised the manuscript. Both authors contributed to the study and have approved the final manuscript.

## Conflict of Interest

The authors declare that the research was conducted in the absence of any commercial or financial relationships that could be construed as a potential conflict of interest.

## Publisher’s Note

All claims expressed in this article are solely those of the authors and do not necessarily represent those of their affiliated organizations, or those of the publisher, the editors and the reviewers. Any product that may be evaluated in this article, or claim that may be made by its manufacturer, is not guaranteed or endorsed by the publisher.
